# The 2024 ISCB Accomplishments by a Senior Scientist Award—Dr Tandy Warnow

**DOI:** 10.1093/bioinformatics/btae289

**Published:** 2024-06-28

**Authors:** Mallory L Wiper

**Affiliations:** The International Society for Computational Biology


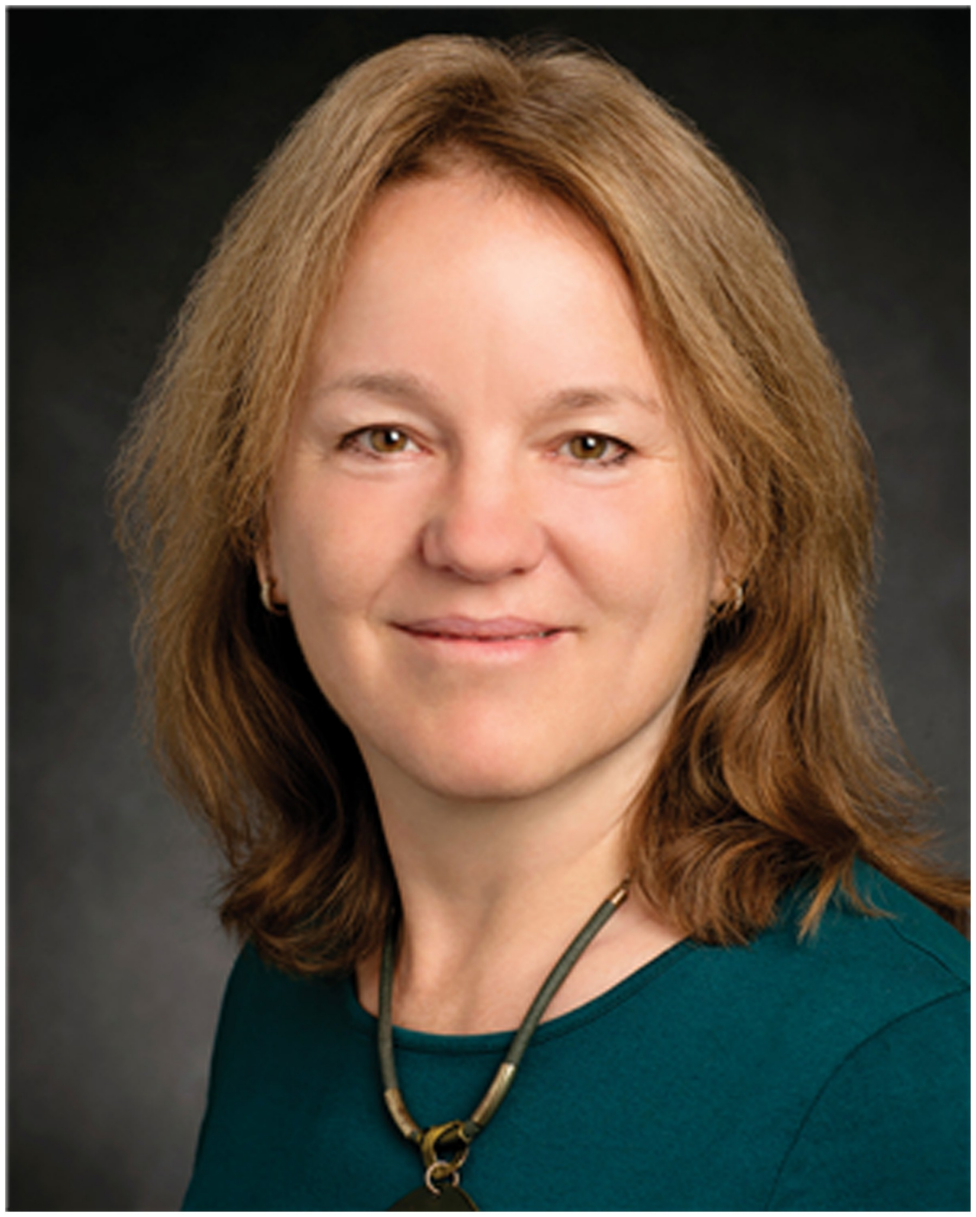


This year, at the 32nd annual conference on Intelligent Systems for Molecular Biology (ISMB) being held in Montreal, Quebec, Canada, the International Society for Computational Biology (ISCB) is proud to be presenting the Accomplishments by a Senior Scientist Award to Dr Tandy Warnow in recognition of her significant contributions to the ever-growing field of computational biology.

## Early inspirations and influences

From a young age, early in her elementary school days, Dr Warnow has loved math. For her, it was something wonderful, fun, and beautiful. She very distinctly remembers a moment from her childhood where the flame of this curiosity was fanned: A teacher talked about counting using 10 as the base simply because that’s how many fingers we have. But what if we had 12 fingers, or six? Instead of being “10” years old in base ten, she could be “14” years old in base six! She’d be a teenager already!

Warnow recalls this moment, and the questions that bubbled to mind, as a “wonderful, fun thing” that really helped her to discover just how cool math could be.

As she got older, the lessons about asking questions and being curious about the world stayed with her. Over time, her education-related interests expanded to include science—with a particular interest in black holes during high school—and the many topics covered therein. But even though science fascinated her, math was still her favorite subject, the love for which continued to be nurtured by her teachers; specifically, by her 9th-grade algebra teacher, Mr Bernstein, who she still remembers with great respect. What Warnow liked most about Mr Bernstein was that he was tough, but fair, expecting a lot from his students and encouraging them to solve problems they thought they couldn’t. He pushed them to do well in a subject that people often found difficult, and Warnow appreciated the intellectual challenge and the belief that he had in his students to do well.

Support from teachers and mentors turned out to be a defining feature during Warnow’s academic career. Many professors during her undergraduate years believed in her and provided an educational environment that fostered her love of math and science, as well as the growth and exploration of the limits of her own understanding.

When she moved into graduate study, continuing her journey with mathematics, her perspective of the *types* of questions math could answer was shifted when her PhD advisor, Gene Lawler, introduced her to algorithmic problems in evolutionary biology, that had what Warnow called “beautiful graph theory versions.” This pivot in perspective continued during her years as a post doc where her advisors, including Michael Waterman, were continuing with this direction of analysis and examination, focusing on the important questions in biology that math could address, and emphasizing utility in biology over mathematical elegance and beauty.

## Research and evolving pursuits

As a Principal Investigator (PI) heading her own lab, Dr Warnow continued to focus on theory from the point of view of her training as a mathematician. That is, until she hired Ken Rice as a post doc, an unexpected inspiration to a new direction for her research questions. Rice introduced Warnow to simulations and the importance of a data-driven approach to big questions. When Warnow transitioned from a theory-only approach and began analyzing data more thoroughly, she discovered that the insights that came to light were very different than those uncovered when working solely within a theoretical framework.

With this new perspective, Warnow’s focus changed to encompass theory *and* experimentation, as the necessity of combining the approaches to gain a comprehensive understanding of a problem and its potential answers had become abundantly clear. Such exploration led her to turn her attention to phylogenetic tree estimation from a theoretical and data-driven point of view.

Some of what fascinated Warnow about this area were the differences in outcomes between theory and data. Focusing only on theory meant that predictions of the accuracy of phylogeny estimation methods, for example, were sometimes based on subtly incorrect interpretations of the theory. Incorporating simulations of evolution allows for a refinement of theoretical frameworks and better understanding of the theory itself. This change in how she approached her work, by combining both theory and simulations, led to a breakthrough in her understanding of methods, models, and theory—and has changed how she approaches all her research questions.

Warnow’s current research is related to phylogenetic networks and other evolutionary questions that extend beyond a simple tree (e.g. hybridization, horizontal gene transfer)—areas she’s found particularly fascinating because of the rarity of feasible tools for analyzing this kind of biological data. In her work with these subjects, she’s discovered how difficult it is to review and interpret such data, which has highlighted a large gap between aspiration and reality in this realm of study. But this gap, and the unanswered questions found there, are a large part of her inspiration to forge ahead with this work!

## Training and mentoring new scientists

Warnow’s shift in status from PhD to post doc to PI has brought about an expansion of her focus and responsibilities to not only include unexplored research questions, but to incorporate the “administrative” side of science as well, like the process of securing grants and navigating the politics of academic publication.

Beyond those tasks, however, her approach to training and mentoring students has been significantly shaped by the meaningful interactions with inspirational people in her academic career, and by her own research interests and experiences. For instance, following her own experience with seeing the importance of combining theory and data to achieve more thorough, cohesive answers to research questions, she’s adamant that students in her lab understand they can’t only focus on theory; they have to incorporate data, too.

When speaking about mentoring students, Warnow stated that “mentoring relationships work [best] when there’s trust in both directions.” As a PI and mentor to those in her lab, she aims to be a support system for students to help them learn to be researchers. She described an experience she had as an undergrad when Turing Award winner Dick Karp talked about the ups and downs of research, and how he weathered the discouraging moments. She shares these lessons with her students, encouraging them to keep going, and to focus on how much fun research is, rather than focusing on trying to be successful. We should not, as researchers, equate poor research outcomes with who we are as people. It is just not true! To help them avoid getting caught in that negative headspace, she empowers her students to believe in themselves and their research and helps them develop coping strategies to handle setbacks.

Above all, just as she received during her formative research years, she supports and believes in her students. She teaches them to ask good questions and to learn to interpret data effectively. Importantly, she also holds high expectations for them, mentoring her students toward mastery.

## Reflections on the Accomplishments by a Senior Scientist Award

Finding out that she was the recipient of the 2024 Accomplishments by a Senior Scientist Award, Dr Warnow said, “I was really shocked.” She had not thought she would be considered for this award, given the growing popularity of machine learning as a tool for problem-solving; a tool upon which her research does not focus. She was quite certain she would not have been in the running since her current focal area, phylogenetics, is not a central research area within the larger field of computational biology but an outlier.

Though she was not expecting it and was truly surprised by the recognition, ISCB is more than pleased to be honoring her with the award this year!

